# Artificial Intelligence Versus Manual Measurement of Spinopelvic Sagittal Parameters Across Preoperative, Postoperative, and Instrumented Spines: A Systematic Review of Diagnostic Accuracy, Failure Rates, and Inferred Surgical Planning Implications

**DOI:** 10.7759/cureus.116863

**Published:** 2026-09-24

**Authors:** Evelyn Abril Jiménez Méndez, Elkin Armando Gallo Tafur, Rafael E Martinez Imbett, Edison Alejandro Hablich Navarrete, Luis Honorio Velez Alarcon, Mishell Alejandra Palacios Cedeño, Antonio Cueva Pérez

**Affiliations:** 1 Neurology, Instituto de Estudios Superiores de Chiapas, Tuxtla Gutiérrez, MEX; 2 Medicine Department, Fundación Universitaria de Ciencias de la Salud, Bogotá, COL; 3 Neuroradiology, Massachusetts General Hospital, Boston, USA; 4 Healthcare Management and Administration, Private Practice, Florida, USA; 5 Tropical Medicine, Ministerio de Salud Pública, Guayaquil, ECU; 6 Medicine, Ministerio de Salud Pública, Montecristi, ECU; 7 General Practice, Universidad Autónoma de Guadalajara, Guadalajara, MEX

**Keywords:** automated measurement, diagnostic test accuracy, machine vision, sagittal balance, spinal deformity correction, spinopelvic parameters, surgical planning

## Abstract

Adult spinal deformity (ASD) demands precise radiographic quantification of spinopelvic parameters to guide corrective surgery. Artificial intelligence (AI) and machine learning (ML) tools offer automated alternatives to manual measurement. This systematic review evaluates the diagnostic accuracy, failure rates, and inferred surgical planning implications of AI-based spinopelvic measurement versus manual assessment, with particular focus on postoperative and instrumented spines. This Preferred Reporting Items for Systematic Reviews and Meta-Analyses (PRISMA) 2020-compliant systematic review searched MEDLINE (PubMed), ScienceDirect, Cochrane Library, and Google Scholar (first 200 results) through June 2026. Studies comparing AI/ML tools, including convolutional neural networks (CNNs), U-Net, Mask R-CNN, and Keypoint R-CNN, against manual clinician measurement on radiographs or EOS biplanar systems were included. Quality was assessed using QUADAS-2. Narrative synthesis was performed given heterogeneity across architectures, populations, and outcome metrics. Sixteen studies (sample sizes ranging from 21 to 1,810 patients per study) compared machine vision against manual measurement across preoperative, postoperative, and instrumented cohorts. Compared to manual measurement, automated machine vision demonstrated agreement statistically comparable to manual measurement for sagittal vertical axis (SVA) (intraclass correlation coefficient (ICC) 0.99-1.00) and pelvic tilt (PT) (ICC 0.96-0.99), matching experienced clinician inter-rater reliability. For lumbar lordosis, automated systems achieved acceptable accuracy in preoperative populations (ICC 0.91-0.97) but exhibited directional biases absent in manual measurement (up to 6.31° overestimation). Thoracic kyphosis and sacral slope agreement was highly variable and dataset-dependent (ICC ranging from 0.58 to 0.97 across studies), generally among the least consistent parameters rather than uniformly weak. Unlike manual measurement, automated accuracy declined significantly in postoperative and instrumented spines. Machine vision failure rates ranged from 0% to 76.2%, versus near-zero manual failure rates. Processing speed was up to 238-fold faster than manual workflows. Automated-versus-manual discrepancies for LL and PI occasionally exceeded the 10° PI-LL surgical correction threshold used as a proxy for surgical-planning impact, since no included study measured a surgical outcome directly. Machine vision matches manual speed and accuracy preoperatively but is not yet reliable enough postoperatively to replace manual clinical assessment without standardised validation.

## Introduction and background

The term "adult spinal deformity" (ASD) refers to a wide range of anomalies of the thoracolumbar or lumbar spine that affect adult patients. Primary degenerative sagittal imbalance, iatrogenic spinal deformity, and adult spinal scoliosis are among the specific ASD diagnoses [1,2]. The incidence and prevalence of ASD are rising due to better medical treatment, longer life expectancies, and a greater percentage of healthy senior adults [1]. According to reports, the incidence of ASD varies from 2% to 32%, and up to 68% of older people have ASD [3,4].

Global sagittal alignment refers to the overall balance of the spine over the pelvis and lower extremities and is distinct from the individual spinopelvic parameters used to quantify it. Since corrective strategies in ASD are guided by validated thresholds, such as a PI-LL mismatch exceeding 10° and an SVA exceeding 40 mm under the SRS-Schwab classification, accurate radiographic quantification of spinopelvic alignment, including pelvic incidence (PI), pelvic tilt (PT), sacral slope (SS), lumbar lordosis (LL), thoracic kyphosis (TK), and the sagittal vertical axis (SVA) [5,6]. PI is a fixed anatomical parameter unique to each patient, while PT and SS are positional parameters that together sum to PI; LL and SVA, in turn, describe how the spine compensates above the pelvis to maintain a level gaze and horizontal pelvis. Because surgical and classification thresholds are defined at fixed cut-points (e.g., PI-LL mismatch >10°, SVA >40 mm), a measurement discrepancy of only a few degrees or millimetres can be sufficient to shift a patient from one side of a threshold to the other, thereby altering deformity classification, surgical candidacy, or the magnitude of correction planned - making even small inter-method differences potentially consequential in a way that would not be true for a continuous outcome without a decision threshold.

Traditionally, clinicians have used digital radiography software like SurgiMap, PACS, sterEOS, or mediCAD to manually assess these parameters [7]. Although manual measurement is regarded as the reference standard, it takes a lot of time, necessitates specialized software training, and is prone to inter- and intra-observer variability that rises with rater experience disparity and case complexity, especially in postoperative or instrumented spines [8]. Convolutional neural networks (CNNs), U-Net and its variations, Mask R-CNN instance segmentation, and Keypoint R-CNN landmark-detection architectures are some of the advances in computer vision and deep learning that have made it possible to measure spinopelvic parameters fully or semi-automatedly from plain radiographs and biplanar stereoradiographic systems like EOS over the past ten years [9]. 

The evidence for AI-based spinopelvic measurement has been compiled in three previous systematic reviews, although each one leaves significant gaps. Bishara et al. did not analyze case-level failure rates, postoperative subgroups, or processing efficiency; instead, they limited their search to PubMed and ASD alone [10]. Glaser et al. never benchmarked AI error against the human inter-/intra-rater variability found in the same source research; instead, they reported mean absolute error and correlation coefficients separately through meta-analysis [11]. The largest breadth (34 studies including all ages and conditions) was provided by Vrtovec and Ibragimov, but their synthesis was limited to architectural description, leaving out failure rates, surgical-status subgroups, and any connection between measurement difference and clinical decision threshold [12]. None of the three examined whether automated accuracy declines disproportionately in postoperative or instrumented spines relative to stable human performance, nor whether reported automated-versus-manual differences are large enough to alter a patient's classification at established surgical correction thresholds. The present review therefore addresses a gap that the existing literature has left largely unresolved: prior reviews have concentrated predominantly on aggregate measurement accuracy, but have not consistently examined case-level failure as a discrete outcome, have not systematically distinguished preoperative from postoperative/instrumented spine performance, and have not benchmarked automated error directly against same-study human reproducibility or against established clinical decision thresholds. The present review fills these gaps directly: tabulating failure rates as a discrete outcome, contrasting pre- and postoperative accuracy, systematically benchmarking AI error against same-study human reliability rather than absolute thresholds, and cross-referencing discrepancies against clinical correction thresholds.

## Review

Methodology

Protocol and Registration

This systematic review was conducted in accordance with the Preferred Reporting Items for Systematic Reviews and Meta-Analyses (PRISMA) 2020 guidelines [13]. A review protocol was not registered prospectively on PROSPERO prior to data collection, and this is acknowledged as a limitation.

Eligibility Criteria

Studies were included if they met the following predefined criteria based on the PICOS framework. Eligible study populations consisted of patients undergoing radiographic assessment of the spine for any indication, including spinal deformity, degenerative disease, or post-surgical evaluation, with no restriction on age, sex, or anatomical region. This deliberately broad population definition was adopted so that the review could capture the full available evidence base on automated spinopelvic measurement, including non-deformity and post-surgical cohorts (e.g., cervical anterior cervical discectomy and fusion (ACDF)/cervical disc arthroplasty (CDA) series). The intervention of interest was any artificial intelligence (AI) or machine learning (ML) tool, including convolutional neural networks (CNNs), instance segmentation models, keypoint detection pipelines, or fully convolutional networks, applied to spinal radiographs or biplanar stereoradiographs (such as EOS) for the automated measurement of sagittal or coronal spinopelvic parameters. Eligible comparators were manual measurements performed by trained clinicians (surgeons, radiologists, or residents) using standard clinical software (e.g., SurgiMap, PACS, sterEOS, mediCAD). Eligible outcomes included diagnostic accuracy metrics, such as intraclass correlation coefficient (ICC), mean absolute error (MAE), root mean square error (RMSE), Pearson correlation coefficient, Bland-Altman analysis, or segmentation performance metrics (Dice similarity coefficient, average precision). Secondary outcomes included processing speed, success/failure rate, and any reported impact on surgical planning. Eligible study designs included retrospective or prospective diagnostic accuracy studies, algorithm development and validation studies, and cohort studies with a human comparator arm. Studies were excluded if they involved three-dimensional CT or MRI segmentation without plain radiograph validation, focused exclusively on non-spinal anatomical regions, lacked a human measurement comparator, were published only as conference abstracts without full-text data, or were written in a language other than English. Non-English records were identified and excluded at the full-text stage on this basis; it is acknowledged that, because a meaningful share of this literature originates from East Asian and European groups, some relevant non-English-language work may exist outside the captured evidence set, and this is noted as a limitation rather than solely as a mechanical exclusion criterion.

Information Sources and Search Strategy

A systematic electronic database search was conducted across four sources: MEDLINE (via PubMed), ScienceDirect, the Cochrane Library, and Google Scholar, from database inception to 23 JUNE 2026, with no date restrictions applied at the search stage.

In PubMed, the following search string was applied: ("artificial intelligence" OR AI OR "machine learning" OR "deep learning" OR "computer vision" OR "machine vision" OR automated OR algorithm*) AND ("sagittal alignment" OR "sagittal balance" OR "spinopelvic alignment" OR "spinal alignment" OR "sagittal vertical axis" OR "pelvic incidence" OR "lumbar lordosis") AND ("spinal deformity" OR "adult spinal deformity" OR scoliosis OR kyphosis OR "spine surgery") AND ("diagnostic accuracy" OR "diagnostic performance" OR reliability OR reproducibility OR validation OR "surgical planning").

In ScienceDirect, the following string was applied across title, abstract, and keyword fields: ("artificial intelligence" OR "machine learning") AND ("sagittal alignment" OR "sagittal balance") AND ("spinal deformity" OR scoliosis) AND (validation OR accuracy).

The Cochrane Library was searched using the following string, applied across title, abstract, and keyword fields: ("artificial intelligence" OR "machine learning" OR "deep learning" OR AI OR algorithm*) AND ("sagittal alignment" OR "spinal alignment" OR "spinopelvic alignment" OR "sagittal vertical axis" OR "pelvic incidence" OR "lumbar lordosis") AND ("spinal deformity" OR scoliosis OR kyphosis OR "spine surgery").

Given the known limitations of conventional database indexing for rapidly evolving AI and engineering literature, a supplementary search of Google Scholar was also performed in accordance with PRISMA guidance on comprehensiveness of retrieval. The following search string was entered into Google Scholar: “automated measurement versus manual measurement sagittal alignment spinal deformity deep learning diagnostic accuracy”.

The first 200 results, sorted by relevance, were screened at the title and abstract level by two independent reviewers, consistent with the methodological approach recommended for grey literature and supplementary Google Scholar searches in systematic reviews. This threshold was applied because the relevance of Google Scholar results decreases substantially beyond the first 200 records when a structured keyword string is used. No date restriction was applied to this search, so recently published studies remained eligible for capture; however, because Google Scholar's relevance ranking is not chronological, it is acknowledged as a limitation that this arm of the search may not have surfaced very recent, lower-citation studies as reliably as an explicit date-sorted check would have. Reference lists of all included studies and of relevant existing systematic reviews identified during screening were also hand-searched, and, for each of the 16 included studies and the three prior systematic reviews, the "Related articles"/"Similar articles" list beneath the corresponding record was manually screened to identify any additional eligible records not captured by the electronic searches.

Study Selection

All retrieved records were exported to a reference management tool and deduplicated. Two independent reviewers screened titles and abstracts against the eligibility criteria, followed by full-text review of all potentially eligible studies. Disagreements at either stage were resolved through discussion and, where necessary, arbitration by a third reviewer; formal inter-rater agreement statistics (e.g., Cohen's kappa) were not calculated for the screening stage, which is acknowledged as a limitation. The selection process and reasons for exclusion at full-text stage were recorded and are summarised in a PRISMA flow diagram (Figure 1).

**Figure 1:**
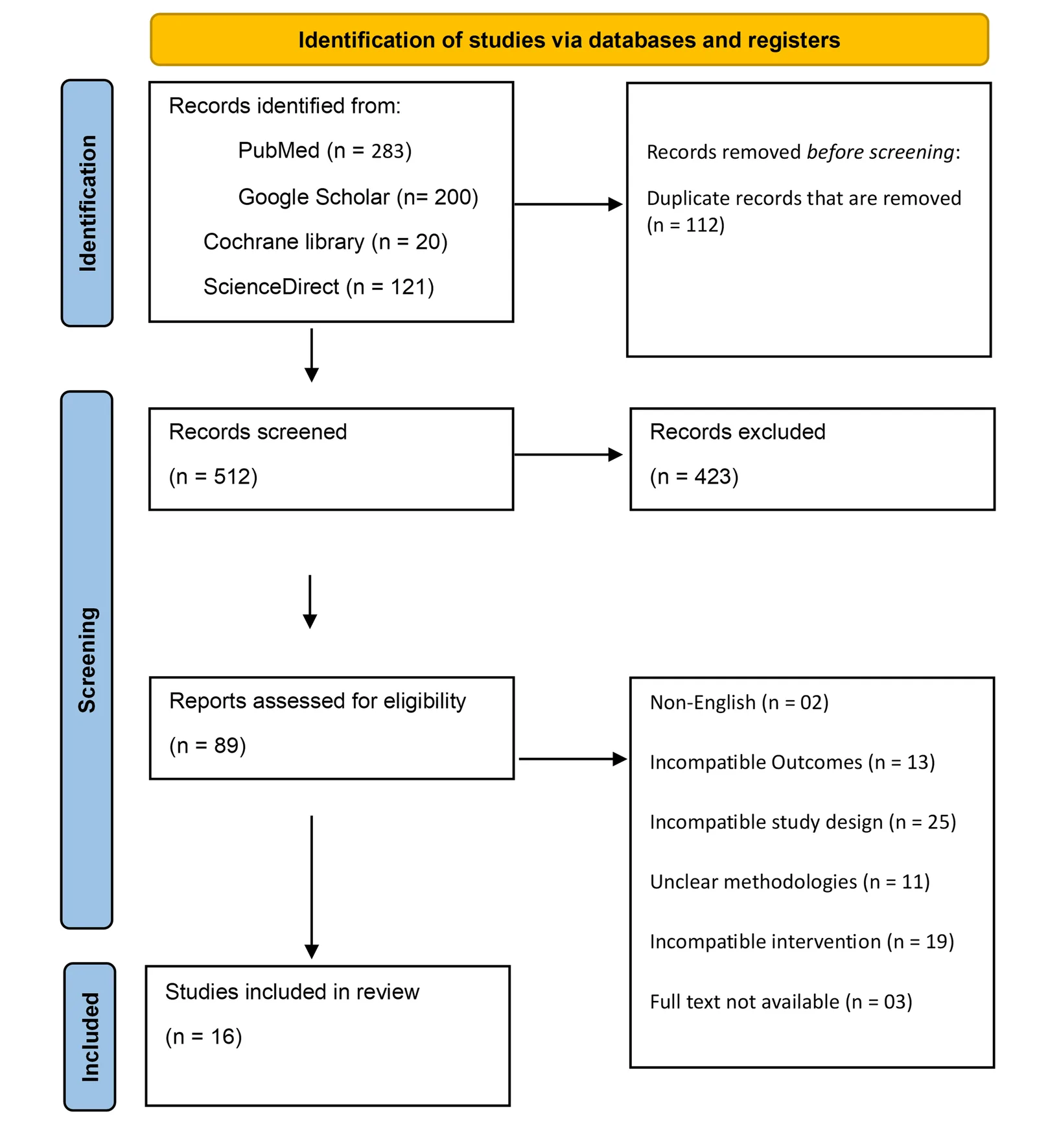
Preferred Reporting Items for Systematic Reviews and Meta-Analyses (PRISMA) flow diagram

Data Extraction

A standardised data extraction form was piloted and applied independently by two reviewers, with discrepancies resolved by consensus. Extracted variables included study design and institution, population characteristics, AI/ML method and architecture (e.g., U-Net, Mask R-CNN, Keypoint R-CNN, ResUNet, RetinaNet), imaging modality, spinopelvic parameters measured, comparator and reference standard, segmentation metrics (Dice, AP50/AP75, or percentage correct keypoints, depending on the architecture), diagnostic accuracy metrics (ICC, MAE, RMSE, Pearson r, Bland-Altman bias/limits of agreement), case-level and parameter-level success/failure rates, processing speed, and pre-/postoperative subgroup data where available. Because source studies defined "failure" inconsistently, failure was operationally standardised as any case where the automated pipeline could not produce a usable measurement without manual correction or exclusion; each study's original definition is retained in the Appendix so this standardisation can be independently checked. Ambiguous, incomplete, or figure-only data were noted during extraction rather than estimated.

Quality Assessment

The methodological quality of each included study was assessed independently by two reviewers using the QUADAS-3 tool, which evaluates four domains: participants (patient selection and sampling), index test (the automated AI/ML measurement pipeline), target condition (adequacy of the reference standard against which the index test was compared), and analysis (whether cases were appropriately included in, or justifiably excluded from, the reported accuracy estimate) [14]. Concerns regarding applicability were also rated for each domain. Disagreements were resolved through discussion. To complement this risk-of-bias assessment, the completeness of AI-specific methodological reporting in each study was independently evaluated using the CLAIM checklist, covering items such as training/validation/test partition sizes, multi-site data provenance, data-augmentation procedures, patient-level split confirmation, external validation, and algorithm-version reporting [15].

Synthesis and Analysis

Given the substantial heterogeneity anticipated across AI architectures, spinal regions, patient populations, and outcome metrics reported, a narrative synthesis was pre-specified and adopted as the sole analytical approach. Statistical meta-analysis was not performed, owing to variability in reference standards (single surgeon versus multi-observer consensus), human comparator experience levels, and the inconsistent reporting of overlapping but non-equivalent accuracy metrics (ICC, MAE, RMSE, Dice similarity coefficient, average precision, percentage of correct keypoints) across studies. No quantitative pooling was performed for any parameter, including SVA and PT, despite these showing tightly clustered ICC values (0.96-1.00) across four or more studies; a Fisher z-transformation random-effects pooling was considered but not undertaken, and is noted as a possible direction for future update of this review.

Findings were synthesised thematically along four axes: anatomical region and parameter type; AI architecture and methodological generation; surgical status of the imaged cohort (preoperative versus postoperative/instrumented); and outcome domain, treating diagnostic accuracy, failure rate, and processing speed as distinct axes.

Where three or more studies reported a comparable metric (principally ICC) for the same parameter (LL, PI, PT, SS, SVA, and TK), these values were benchmarked descriptively against the corresponding human inter-rater or intra-rater reliability reported in the same study, so automated-versus-manual agreement was judged relative to pre-existing manual measurement variability rather than an arbitrary absolute threshold. This benchmarking compared point estimates as reported by each source study; no formal statistical test of the AI-versus-human error difference was performed, and this is noted as a limitation.

Processing speed and failure rate were synthesised separately as secondary outcomes. Surgical planning impact was assessed by cross-referencing discrepancies against established correction thresholds (PI-LL >10°, SVA >40 mm). Heterogeneity sources were explored qualitatively; reporting bias was considered narratively, as no funnel-plot analysis was feasible without pooled estimates.

Certainty of Evidence

A formal certainty-of-evidence framework (e.g., GRADE) was not applied in this review. This decision was made because GRADE is designed primarily for pooled effect estimates across comparable outcomes, and the majority of parameters in this review were synthesised narratively rather than quantitatively; certainty of evidence is instead addressed qualitatively, per parameter, by considering risk of bias (QUADAS-3), consistency of direction of effect across studies, and reference-standard comparability (assessed via the Target Condition domain).

Results 

A systematic database search identified 624 records across four databases: PubMed (n = 283), Google Scholar (n = 200), Cochrane Library (n = 20), and ScienceDirect (n = 121). Following the removal of 112 duplicate records, 512 records were screened by title and abstract, of which 423 were excluded. The remaining 89 reports were assessed for full-text eligibility, and 73 were subsequently excluded for the following reasons: incompatible study design (n = 25), incompatible intervention (n = 19), incompatible outcomes (n = 13), unclear methodologies (n = 11), non-English language (n = 2), and full text not available (n = 3). Following this screening process, 16 studies met all eligibility criteria and were included in the final qualitative synthesis. The selection process is illustrated in the PRISMA flow diagram (Figure 1).

Study Characteristics

Sixteen studies met the inclusion criteria, spanning adult and elderly populations with adolescent idiopathic scoliosis, ASD, degenerative lumbar and cervical disease, and post-surgical instrumented spines. Sample sizes ranged from 21 patients to 1,810 unique patients contributing 2,007 radiographs, with several studies reporting separately on total radiograph counts that exceeded patient counts due to repeated or multi-view imaging. The imaging modality was predominantly standing lateral or whole-spine radiography, with three studies using biplanar EOS stereoradiography. Spinal regions evaluated included the lumbar spine, the cervical spine, and the whole spine from the cervicothoracic junction to the pelvis. Four studies assessed both preoperative and postoperative images, and instrumented or post-surgical cohorts were specifically represented in five studies. AI architectures ranged from single-task CNN/U-Net pipelines in earlier studies to multi-stage instance segmentation and keypoint-detection networks in later studies, including Mask R-CNN, Keypoint R-CNN, and a commercial automated platform. The manual comparator was consistently one or more fellowship-trained spine surgeons, attending neurosurgeons, radiologists, or a multi-observer consensus panel using standard clinical measurement software such as SurgiMap, PACS, sterEOS, or mediCAD. The parameters most frequently compared between automated and manual methods were lumbar lordosis, pelvic incidence, pelvic tilt, sacral slope, sagittal vertical axis, thoracic kyphosis, and Cobb angle. These characteristics are summarized in Table 1.

**Table 1:** Characteristics of the studies included. Abbreviations: AI = artificial intelligence; CNN = convolutional neural network; CV = computer vision; DSNT = differentiable spatial to numerical transform; EOS = EOS imaging; FPN = feature pyramid network; LL = lumbar lordosis; NR = not reported; PI = pelvic incidence; PT = pelvic tilt; SVA = sagittal vertical axis; SS = sacral slope; TK = thoracic kyphosis

Author(s) and year	Study design	Population and N	Mean age (years)	AI/ML intervention	Control / comparator
Cho et al., 2020 [16]	Cross-sectional database study	Lateral lumbar X-rays; training/validation n = 629, test n = 151; surgeon comparison n = 50	NR	U-Net CNN for vertebral segmentation with CV-based LL measurement; image augmentation; Keras/Python	Three surgeons; attending surgeon as reference standard; repeated measurements
Korez et al., 2020 [17]	Retrospective diagnostic accuracy study	55 adults, 97 sagittal X-rays; included pre/postoperative images	69.6 (35–85)	Two CNNs: RetinaNet for anatomical detection and U-Net for landmark regression; measured SS, PT, PI, ST, and SSA	One spine surgeon using SurgiMap; manual reliability and time assessed
Kim et al., 2023 [18]	Retrospective diagnostic accuracy/pipeline study	1,810 patients; 2,007 radiographs; test n = 200	NR	Mask R-CNN with ResNeXt-101-FPN plus CV processing; measured TK, TLK, LL, SS, PT, PI, and SVA	Three surgeons; senior neurosurgeon as criterion standard
Zerouali et al., 2023 [19]	Retrospective observational study	100 patients; whole-spine standing radiographs; test set n = 90	Median 14 (3–64)	Mask R-CNN with ResNet50-FPN; three model variants; multiple coronal and sagittal parameters	Junior radiologist measurements reviewed by a senior MSK radiologist; specialist/resident comparison
Vogt et al., 2024 [8]	Retrospective monocentric validation study	129 patients undergoing anterior cervical surgery; training data from 18 sites	53	Four interlinked CNNs comprising Mask R-CNN and U-Nets; assessed cervical alignment parameters	Two experienced physicians; blinded repeated measurements
Almahozi et al., 2025 [20]	Retrospective single-center validation study	21 adults with degenerative spinal disease; instrumented and non-instrumented	NR	Brainlab Elements automated vertebral labeling and spinopelvic parameter measurement from EOS images	Eight observers using PACS; two measurement rounds
Caesarendra et al., 2022 [21]	Diagnostic accuracy study using public and local data	AIS; 551 X-rays; train n = 481, test n = 70	NR	ACAMM using 152-layer ResNet CNN; automated vertebral landmark detection and Cobb angle calculation	Two neurosurgeons; scoliosis specialist as reference standard
Hoppe et al., 2025 [22]	Retrospective multi-reader diagnostic accuracy study	65 test patients with lateral EOS radiographs; fractures and instrumentation included	47.8 ± 24.1 (7–85)	Two-stage R-CNN for vertebral centers and corner landmarks; measured TK, LL, SS, and SVA	Six radiologists/orthopedic surgeons; median reader measurement as reference
Weng et al., 2019 [23]	Retrospective algorithm development/validation	990 patients; 99-image reader subset; whole-spine lateral radiographs	Age-stratified; mean NR	31-layer ResUNet for C7 and sacral landmark detection; SVA calculation	Three human annotators; senior resident labeling used for full dataset
Yuan et al., 2024 [24]	Retrospective model development/validation	1,240 adult lateral lumbar radiographs; train 992, validation 124, test 124	52.46 ± 16.54 training; 51.79 ± 17.23 test	RADMFNet with dilated convolution, ResNet, and attention-based feature fusion; LL and pelvic parameters	Three spine surgeons; mean of surgeon measurements as reference
Orosz et al., 2022 [25]	Retrospective validation study	100 lumbar fusion patients; 200 validation radiographs; 600 training images	NR	Cascade R-CNN plus 2 U-Net models for vertebrae, sacrum, femoral heads, and instrumentation; LL, PI, PT, and SS	Two spine surgeons; blinded AI and human measurements
Harake et al., 2024 [26]	Retrospective model development/validation	761 training/validation and 40 test whole-spine radiographs; high prevalence of deformity/instrumentation	NR	SpinePose using three Keypoint R-CNN models; measured SVA, PT, PI, SS, LL, and pelvic parameters	Four reviewers, including spine surgeons, a radiologist, and a resident
Löchel et al., 2024 [27]	Retrospective validation study	141 ASD patients; 243 pre/postoperative images; long-construct instrumentation	NR	Three-stage Mask R-CNN and U-Net pipeline for segmentation, landmark detection, and spinopelvic measurements	Two human raters using SurgiMap; repeated measurements
Lang et al., 2024 [28]	Retrospective diagnostic accuracy study	393 patients; 555 EOS bi-planar radiographs; AIS and ASD	42.2 ± 24.3	Modified U-Net with inception modules; multi-ROI segmentation and landmark detection; multiple sagittal/coronal parameters	Blinded medical expert using mediCAD; additional inter/intra-rater assessment
Lee et al., 2025 [29]	Retrospective diagnostic accuracy study	888 lateral X-rays; test n = 50; general spinal pathology	48.7 ± 24.78 test	Keypoint R-CNN with ResNeXt-101-FPN; automated L1/S1 landmark detection for LL measurement	Two orthopedic surgeons with >10 years' experience
Galbusera et al., 2019 [30]	Retrospective diagnostic accuracy study	493 EOS bi-planar radiographs; AIS, adult scoliosis and degenerative disorders; test n=50	NR	FCN with DSNT; 78 landmark-localizer models for 39 landmarks in 2 planes; multiple alignment parameters	sterEOS 3D reconstruction used as reference standard

Quality Assessment

QUADAS-3: QUADAS-3 domain-level ratings for all 16 studies are shown in Figure 2. Participants were the most frequent driver of high risk, largely reflecting non-consecutive, single-institution sampling (Cho, Korez, Zerouali, Almahozi, Caesarendra, Yuan); Hoppe was the only study with explicitly consecutive sampling. Index Test concerns were also common, with Kim, Yuan, Korez, and Lang rated high risk, generally reflecting limited pre-specification or reporting transparency around the automated pipeline. Target Condition was the best-controlled domain overall, with only Korez (single rater) and Galbusera (non-clinician sterEOS reference, no human comparator) rated high risk; most other studies used two or more raters. Analysis concerns were driven by unincorporated case exclusions - Cho, Kim, Almahozi, Löchel, and Lang all excluded segmentation or detection failures from their headline accuracy figures without reflecting this in reported performance metrics.

**Figure 2:**
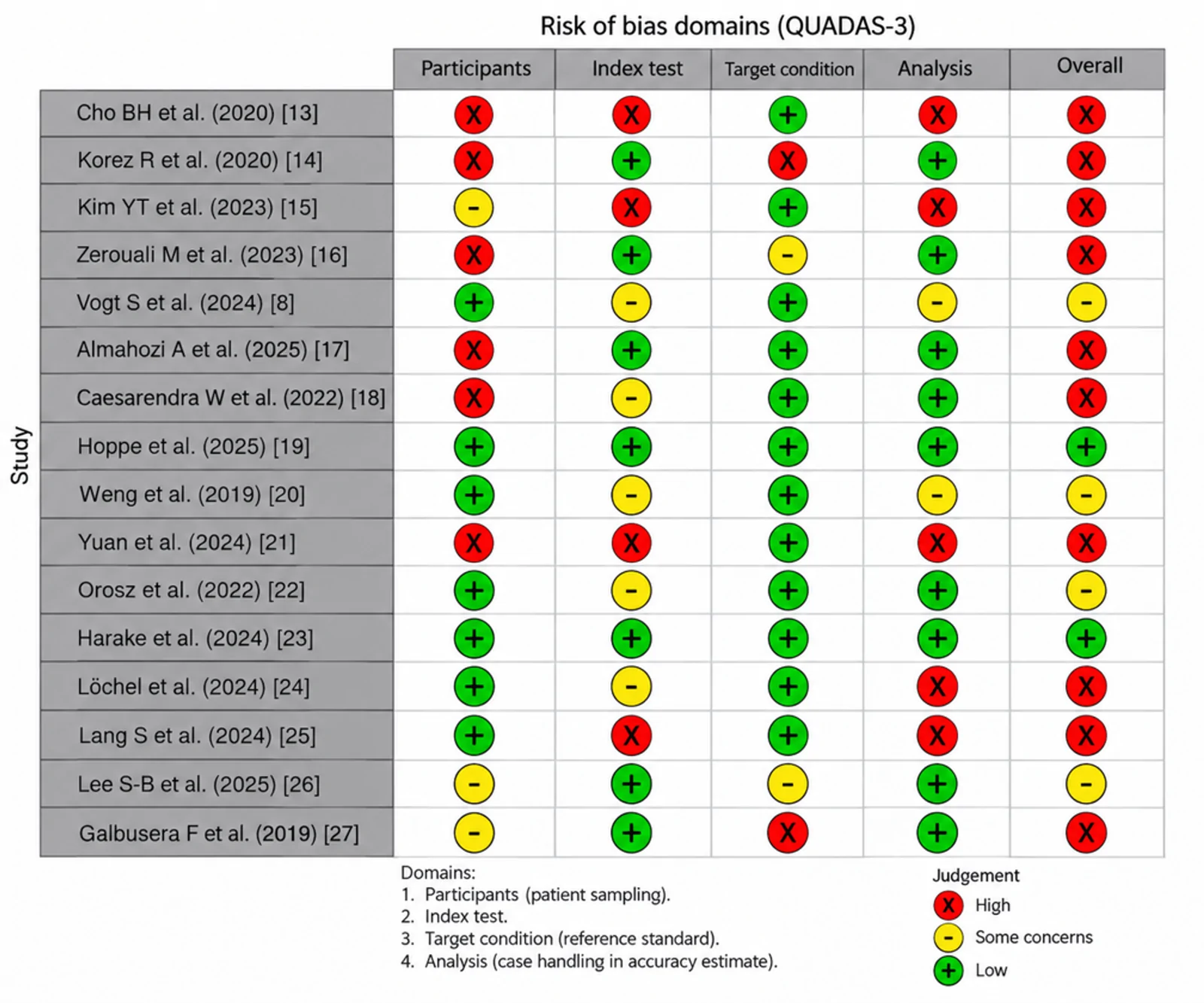
Quality assessment of studies by QUADAS-3.

Overall, two studies (Hoppe, Harake) were rated low risk across all four domains; three studies (Vogt, Orosz, Weng) were rated some concerns; and the remaining eleven studies were rated overall high risk, most often from a combination of non-consecutive sampling and unincorporated case exclusion (Figure 2).

CLAIM: The CLAIM checklist was applied as a complementary reporting-completeness assessment. AI/ML architecture, platform, and measured parameters were reported for all 16 studies. Training/validation/test partition sizes were reported for 13 of 16 studies; multi-site training data were reported for three of 16 (Vogt, Orosz, Löchel - 18, 18, and 22 sites respectively); and explicit data-augmentation procedures were reported for three of 16 (Cho, Lee, Galbusera). Confirmation of patient-level (as opposed to image-level) separation between partitions, formal external-site or cross-scanner validation, and algorithm-version reporting were absent from all 16 studies - the principal reporting gap this assessment identifies.

Sensitivity Analysis Restricted to Lower-Risk-of-Bias Studies (Post Hoc)

As recommended by the peer review, we conducted a post-hoc narrative sensitivity check restricted to the six studies rated low risk of bias (Hoppe et al., Harake et al.) or some concerns (Vogt et al., Weng et al., Orosz et al., Lee et al.) on QUADAS-2, to assess whether this review's core conclusions are driven disproportionately by the 10 higher-risk studies. Within this restricted subset, SVA and PT agreement remained excellent: Hoppe et al. reported an SVA ICC of 1.00 (95% CI 0.99-1.00) with no significant difference from any individual human reader, and Weng et al. reported ICCs of 0.989-0.993 against three independent readers, closely matching inter-rater reliability; Harake et al. similarly found ICCs of 0.91-1.0 for lumbar and pelvic parameters with no significant AI-versus-human difference (p = 0.24-0.93). Postoperative decline was also preserved within this lower-risk subset: Orosz et al., which directly compared preoperative and postoperative cohorts, reported ICCs falling from 0.85-0.92 preoperatively to 0.75-0.87 postoperatively, with a corresponding increase in RMSE, consistent with the full-sample finding of postoperative performance decline. TK could not be fully re-examined within this restricted subset, since only Hoppe et al. reported TK data among the six lower-risk studies (ICC 0.92, no significant difference from human readers); the wide TK variability described across the full 16-study sample is therefore driven substantially by the higher-risk subset, and that specific finding should be interpreted with additional caution. A systematic directional bias was also preserved in this subset despite low risk-of-bias ratings: Lee et al. (some concerns) reported a persistent LL underestimation of −3.42° to −4.28° despite an excellent ICC of 0.918, reinforcing this review's broader argument that a high ICC alone can mask clinically relevant bias even among better-quality studies. Overall, the core conclusions regarding SVA/PT reliability and postoperative decline appear qualitatively robust when restricted to the lower-risk-of-bias subset, whereas conclusions regarding TK are less well supported by this subset alone and rely more heavily on the higher-risk studies. This check is a narrative sensitivity comparison rather than a formal quantitative subgroup analysis or meta-analysis and should be regarded as provisional; a structured, quantitative sensitivity analysis conducted with statistician input is recommended before the review's risk-of-bias robustness can be considered fully established.

Reliability and Failure Rates

Manual measurement in experienced hands is rarely unable to produce an output, since experienced clinicians can apply contextual anatomical knowledge to resolve overlapping structures, artefacts, and transitional anatomy that automated systems may fail to handle. In contrast, automated failure rates in the included studies ranged from 0% to 76.2% of patients having at least one failed parameter measurement. Because source studies defined "failure" using markedly different operational criteria, this range should be interpreted with that heterogeneity in mind; for this review, failure was standardised as any case in which the automated pipeline could not produce a usable measurement without manual correction or exclusion, with each study's original definition retained separately for verification. Almahozi et al. reported the highest overall automated failure burden (16 of 21 patients, 76.2%; 39 of 189 measurements, 20.6%) [19]. Lang et al. found complete automated segmentation in only 45 of 100 test cases (45%), attributing failures to severe pathology, high BMI, and foreign objects [25]. Cho et al. reported a 15.9% failure rate due to inability to identify L1 or S1 [18]; Kim et al. excluded 6% of test cases due to segmentation failures at sacrum-L5 overlap and T4 artefact [17]; Zerouali et al. excluded 10 of 94 evaluation cases [26]. Hoppe et al., Harake et al., Lee et al., and Galbusera et al. all reported 100% case completion [16,20,23,27], indicating failure rates are architecture- and population-dependent rather than an inherent limitation of automation as a category.

Because "failure" was operationalised differently across source studies, the reported figures above are reclassified here, to the extent the extracted data permit, into a four-tier hierarchy: (1) case-level technical failure, no valid automated output for the case at all - best exemplified by Lang et al., where complete automated segmentation was achieved in only 45 of 100 test cases; (2) parameter-level failure, one or more individual parameters unavailable while others succeeded - exemplified by Cho et al. (inability to identify L1 or S1 in 15.9% of cases) and Kim et al. (6% of cases excluded for segmentation failure at the sacrum-L5 overlap or T4 artefact); (3) human-corrected output, algorithmic output requiring manual adjustment before use; and (4) successful-but-clinically unacceptable output, a numerical result generated but falling outside a predefined error threshold. Tiers 3 and 4 could not be reliably reclassified from the extracted text, since most source studies did not report whether flagged or corrected outputs were retained, discarded, or manually adjusted prior to accuracy analysis; this is acknowledged as a limitation of the present synthesis and is flagged for confirmation against the original source papers.

Processing Speed

Automated measurement most consistently and substantially outperformed manual measurement on processing speed. Hoppe et al. reported whole-spine extraction completed in under one second versus a mean manual reading time of 139 seconds across six readers (~139-fold reduction) [23]. Korez et al. found sub-one-second automated processing versus 75 seconds manual, plus a 30-minute software tutorial requirement for manual measurement [21]. Zerouali et al. timed automated multi-parameter extraction at 3.8 seconds versus 732.4 and 904.15 seconds for specialist and junior resident, respectively (192-fold and 238-fold reductions) [26]. Almahozi et al. confirmed a statistically significant 3.6-fold reduction for the commercial Brainlab platform (t(20) = −9.952, p < 0.001) [19]. Cho et al. and Weng et al. reported sub-second per-case computation for single-parameter pipelines [18,24].

The fold-reductions above (up to 238-fold) are, in every case, comparisons of raw algorithmic inference time against manual measurement time; they are not end-to-end workflow comparisons, since none of the included studies' reported figures incorporated PACS integration, image preprocessing, server latency, or model maintenance overhead on the automated side. Critically, none of the included studies measured real-world clinician time after mandatory human verification of automated output, which is the time metric most relevant to actual clinical deployment; the reported speed advantage should therefore be read as an upper bound on efficiency gains rather than a validated estimate of net clinician time saved.

Measurement Agreement: Intraclass Correlation and Bland-Altman Analysis

Statistical agreement between automated and manual measurement, quantified by ICC and Bland-Altman analysis, was the primary metric used across studies. ICC, Pearson's r, and R² are not statistically interchangeable measures of agreement; Table 2 records the specific statistic reported by each source study, and readers are cautioned against direct numeric comparison across rows using different statistics. ICC values ranged from poor-to-moderate in early-generation or complex-population studies to excellent in mature, homogeneous-dataset pipelines. The highest ICCs were consistently achieved for SVA (0.99-1.00) and PT (0.96-0.99) [16,17,23,24,25], both also showing the narrowest Bland-Altman limits of agreement (LoA). The lowest were observed for SS, TK, and PI in complex postoperative cohorts [22,25,26]. Comparisons of "AI error versus human inter-rater variability" throughout this section are, unless stated otherwise, comparisons of point estimates reported side by side within the same study, not the output of a formal statistical test (e.g., a difference-in-differences calculation or confidence-interval overlap check); this is noted as a limitation. Korez et al. found automated SS and PI MAD close to manual intraobserver MAD for the same parameters [21]. Orosz et al. found automated-versus-rater ICC comparable to human inter-rater ICC (0.85-0.92 vs. 0.92-0.96) [29]. Directional biases were detected in several systems - Cho et al. (LL overestimation, 26/42 cases) [18], Lee et al. (LL underestimation, bias −3.42° to −4.28°) [20], Almahozi et al. (LL and SVA overestimation) [19] - biases not present in manual measurement and capable of misclassifying patients relative to surgical thresholds. Full per-study figures for LL, PI, PT, SS, SVA, and TK are provided in Table 2.

**Table 2:** Summary of automated (AI) versus manual measurement agreement for spinopelvic parameters reported by three or more studies. Abbreviations: AP = average precision; AR = average recall; CNN = convolutional neural network; DSC = Dice similarity coefficient; FPN = feature pyramid network; ICC = intraclass correlation coefficient; IoU = intersection over union; LL = lumbar lordosis; MAD = mean absolute deviation; MAE = mean absolute error; mAP = mean average precision; OKS = object keypoint similarity; PCK = percentage of correct keypoints; PI = pelvic incidence; PT = pelvic tilt; R² = coefficient of determination; SVA = sagittal vertical axis; SS = sacral slope; TK = thoracic kyphosis

Study	Segmentation/landmark architecture	Segmentation/landmark performance	Parameter(s) assessed	AI-vs.-reference agreement	Human reliability	Case-level success/completion	Processing time / speed advantage	Clinical relevance / key limitation
Cho et al., 2020 [16]	U-Net CNN (vertebral body segmentation)	Training DSC 0.966; validation DSC 0.923; test DSC 0.821; AUC 0.914; accuracy 0.862	LL	ICC 0.958	ICC 0.933–0.984 (3 surgeons)	127/151 (84.1%) LL successfully measured; ~16% failed due to inability to identify L1/S1 endplate	0.14 s/radiograph; manual time not directly compared	High LL agreement; failures mainly related to L1/S1 endplate identification
Korez et al., 2020 [17]	RetinaNet (bounding box) + U-Net (landmark regression)	Bounding-box/landmark-probability output; DSC not reported	PI, PT, SS	PI: r = 0.81; PT: r = 0.90; SS: r = 0.73	PI MAD 4.3° (SD 5.4°); PT MAD 1.7° (SD 2.5°); SS MAD 4.1° (SD 4.6°)	96/97 (99%) images processed; PI/PT reduced to 90/97 where femoral heads not visible	≤1 s/image vs 75 s manual; ~75× faster (excluding 30-min tutorial)	PT showed strongest agreement; SS weakest; femoral-head visibility limits PI/PT assessment
Kim et al., 2023 [18]	Mask R-CNN (ResNeXt-101-FPN)	AP50 96.2%; AP75 75.7%; overall AP 64.9%; AR 70.4%; DSC 92.6%; class AP50 54.8–81.5%	LL, PI, PT, SS, SVA, TK	LL 0.95; PI 0.95; PT 0.99; SS 0.86; SVA 0.99; TK 0.97	LL/PI ICC 0.94; PT ICC 0.99, MAE 0.7°	188/200 (94%) retained; 6 segmentation failures + 6 sacralization/lumbarization cases excluded	Not reported	Excellent agreement, particularly PT/SVA; failures associated with artefact, sacral overlap, and transitional anatomy
Zerouali et al., 2023 [19]	Mask R-CNN (ResNet50-FPN), best variant HL512	Detection AP0.75 0.73; segmentation AP0.75 0.706; IoU threshold 0.80	LL, PI, PT, SS, TK	LL 0.76; PI 0.78; PT 0.85; SS 0.62; TK 0.58	Not directly isolated; Bland–Altman bias TK 4.21° vs. 2.57° resident	72/100 (72%) reliable without modification	3.8±0.8 s vs 732.4 s specialist and 904.15 s resident; ~192×/~238× faster	PT performed best; TK and SS showed weaker agreement; lower reliability and failed cases limit robustness
Vogt et al., 2024 [8]	Four interlinked CNNs (Mask-RCNN + 3 U-Nets)	Dice 0.91 preoperatively; 0.87 postoperatively	Cervical landmarks / alignment	Not reported as ICC	Not reported	C1/C2/C7 detected in 99% preop and 98% postop; T1 slope only 48–51%; 3 C7 failures	Not reported	Strong cervical landmark detection except T1 slope; postoperative/instrumentation complexity affects performance
Almahozi et al., 2025 [20]	Commercial automated labeling (Brainlab Elements)	Not a segmentation-network study; Dice/AP/PCK not reported	LL, SVA and other sagittal parameters	LL ICC 1.00; SVA ICC 1.00 (intraobserver automated)	Intraobserver 0.44–0.99; interobserver 0.53–0.99	Overall success 79.4%; ≥1 parameter failed in 16/21 patients; 39/189 measurements failed (20.6%)	62±27 s / 58±21 s vs 227±72 s / 221±65 s; ~3.6× faster	Very high agreement for successfully measured parameters, but substantial individual-measurement failure; LL bias up to 6.31°
Caesarendra et al., 2022 [21]	152-layer ResNet CNN (ACAMM)	Correctly detected 17 vertebrae/image; robust to contrast, lighting, body habitus	Vertebral identification / Cobb angle	69/70 (98.6%) within ±5° of reference	Not reported	69/70 (98.6%) within ±5°; 1 failure in >40° Cobb-angle subgroup	GPU inference; numerical time not reported	High robustness across image conditions; reduced accuracy at larger Cobb angles
Hoppe et al., 2025 [22]	Two-stage R-CNN (landmark detection)	Landmark-based; pixel segmentation not primary endpoint	SVA, TK and landmarks	SVA ICC 1.00 (95% CI 0.99–1.00); TK ICC 0.92	Reader MAE: SVA 1.22–2.79 mm; TK 1.97–3.87°	65/65 (100%) cases processed, including fractures, deformity, and instrumentation	≤1 s/case vs 139 s manual; ~139× faster	Excellent SVA agreement and complete case processing; supports use in complex spinal pathology
Weng et al., 2019 [23]	31-layer ResUNet (keypoint heatmap)	5-mm detection: C7 91.1%, sacrum 87.1%; 1-mm detection: C7 49.6%, sacrum 32.8%	SVA / C7 and sacral landmarks	SVA r=0.989–0.993	Inter-rater r = 0.949–0.996	No binary pass/fail; outlier rate >10 mm = 6.87%	~0.2 s/case for SVA calculation	Excellent SVA correlation; sub-5-mm landmark precision considerably lower than 5-mm detection
Yuan et al., 2024 [24]	RADMFNet (dilated convolution + SE-ResNet)	Lumbar Dice 0.962, accuracy 0.947; sacrum Dice 0.954, accuracy 0.939; PCK ≤3 mm 88–98%	LL, PI, and pelvic parameters	LL ICC 0.97	Inter-observer 80–83% (≤3 mm); intra-observer 96%	All images processed; no case-level failure reported	Not explicitly quantified	Excellent segmentation and LL agreement; high landmark accuracy; human comparison metric differs from ICC
Orosz et al., 2022 [25]	Cascade R-CNN + 2 U-Nets	Dice 0.85; anatomical detection rate 96%; landmark Euclidean error 1.9–2.1 mm	LL, PI, PT, SS	LL/PI/PT/SS: 0.85–0.92 preop; 0.75–0.87 postop	Preop ICC 0.92–0.96; postop 0.90–0.95	98% preop, 95% postop; failures due to undetected L1/femoral heads and transitional anatomy	Not explicitly quantified; described as time-saving	Good-to-excellent agreement; postoperative instrumentation and anatomical variation reduce performance
Harake et al., 2024 [26]	SpinePose (three parallel Keypoint R-CNNs)	PCK@5 mm: 75–93% (L1/S1), 38–60% remaining; PCK@10 mm: 88–100%	LL, SVA, and spinal landmarks	0.91–1.00 for reported parameters	Not separately isolated	40/40 (100%) test images produced predictions	Not explicitly quantified	High 10-mm landmark accuracy; lower 5-mm PCK for intermediate vertebrae
Löchel et al., 2024 [27]	Mask-RCNN + U-Net (heatmap regression)	Qualitative segmentation; Dice/AP not reported	LL, PI, PT, SS, SVA	LL 0.71–0.99; PI 0.71–0.85; PT/SS within 0.71–0.99; SVA 0.96–0.99	Preop ICC 0.96–0.99; postop ≥0.99	91.5% preop; 84.8% postop; postoperative failures associated with long-construct instrumentation artefacts	Not explicitly quantified	SVA most reliable; PI showed largest AI-human error (RMSE up to 10.2°); postoperative instrumentation is important limitation
Lang et al., 2024 [28]	Modified U-Net with inception modules	Preprocessing successful in 533/555 (96%); complete multi-ROI segmentation 45/100 (45%)	LL, PI, PT, SS, SVA, TK	LL 0.82; PI 0.70; PT 0.96; SS 0.61; SVA 0.99; TK 0.69	Excellent (>0.9) for reported parameters	45/100 (45%) complete multi-parameter predictions; frontal/Cobb-only success 61%	Not reported	SVA/PT strongest; PI/SS/TK weaker; severe pathology, high BMI, and foreign objects reduced completion
Lee et al., 2025 [29]	Keypoint R-CNN + FPN (ResNeXt-101)	Bounding-box mAP 71.63; keypoint mAP (OKS) 86.61	LL / spinal keypoints	LL ICC 0.918 vs S1 and 0.949 vs S2	ICC 0.962 (all three surgeons combined)	50/50 (100%) test images processed	Not reported	Strong keypoint performance and agreement with surgeons; processing speed not reported
Galbusera et al., 2019 [30]	FCN + DSNT (78 localizer models)	No F1/Dice/mAP; qualitatively perceptually convincing	PI, PT, SS, SVA, TK	PI R² = 0.52; PT R² = 0.89; SS R² = 0.58; TK R² = 0.79	Not directly comparable (sterEOS reference)	50/50 (100%) test cases processed; known failure zones at T1/S1 endplates and coronal S1 orientation	Not reported	PT strongest and PI weakest; limitations related to T1/S1 landmark localization and reference-system differences

Statistical Agreement Versus Clinical Interchangeability

ICC, Pearson correlation, mean (Bland-Altman) bias, and LoA answer different questions and are not interchangeable indicators of clinical readiness. ICC quantifies relative reliability across the sample studied and is sensitive to the between-subject variance of that sample; it does not by itself establish that two measurement methods can be substituted for one another in an individual patient. Mean bias describes the average systematic offset between methods and can be small even when individual-case errors are large. Limits of agreement describe the range within which most individual differences fall and are the statistic most directly relevant to whether a single patient's automated measurement can be trusted without manual verification; a wide LoA does not automatically imply clinical inadequacy unless interpreted against a predefined clinically acceptable error margin, and conversely a small mean bias can coexist with clinically unacceptable individual-case errors. This review's own data illustrate why the distinction matters: several algorithms combine excellent ICC with clinically meaningful Bland-Altman bias and wide limits of agreement (e.g., Cho et al.'s LL pipeline, ICC 0.958 but MAE 8.055° with SD 12.989°; Lee et al.'s persistent LL underestimation of −3.42° to −4.28° despite an ICC of 0.918). A high ICC in these cases reflects consistent relative ranking across the sample, not interchangeability for any given patient's surgical decision-making. Statistical significance testing carries a parallel caveat: a non-significant paired difference (p>0.05) indicates only insufficient evidence of a mean difference under that specific analysis and does not, by itself, demonstrate equivalence; a formal equivalence claim would require a prespecified clinical equivalence margin, which none of the included studies defined. Readers should therefore treat statements of automated-versus-manual "agreement" in this review as claims about reliability and mean-level correspondence, not as claims of clinical interchangeability at the individual-patient level.

Parameter-Specific Performance: Lumbar and Pelvic Parameters

Lumbar lordosis (LL): Automated LL accuracy was generally acceptable, particularly in homogeneous, non-surgical populations. Yuan et al.'s RADMFNet system produced LL values with a mean difference of only 1.69° and an ICC of 0.97 versus the three-surgeon reference [15]. This difference was not significant on t-test (p = 0.58); as with similar findings elsewhere in this review, a single non-significant test in one sample should not be read as a formal demonstration of equivalence. Harake et al. and Kim et al. similarly found no significant difference from human reviewers [16,17]. Cho et al.'s pipeline was not significantly different from manual measurement in mean LL error (p = 0.372) [18]. However, its case-level error variability (SD 12.989°) far exceeded human variability (~3.2°), illustrating that a non-significant mean difference can coexist with clinically unacceptable individual-case spread. Almahozi et al. and Lee et al. both found systematic directional bias despite high ICC [19,20], reinforcing that ICC alone does not preclude clinically meaningful bias.

Pelvic Incidence (PI): PI showed more variable agreement than PT or SVA. Löchel et al. found PI had the largest mean errors of any parameter in their ASD cohort [22]. Korez et al. found no significant differences across all five pelvic parameters (paired t-test p > 0.05) [21]; this non-significant result reflects no detected mean difference in the sample tested, not a formal equivalence demonstration, since no equivalence-margin analysis was reported.

Pelvic tilt (PT): PT was among the most consistently well-performing parameters. Kim et al. reported PT MAE of 0.4°, numerically exceeding inter-surgeon agreement [17]; Korez et al. found automated PT MAD close to manual intraobserver variability [21].

Sacral slope (SS): SS was among the weaker lumbar/pelvic parameters, with Kim et al., Zerouali et al., and Lang et al. all reporting it as their weakest or near-weakest parameter [17,25,26].

Thoracic, Cervical, and Coronal Parameters

TK showed the most variable agreement of any sagittal parameter. Hoppe et al. found no significant difference from any individual human reader (post-hoc Tukey) [23], one of the few instances where a formal statistical test, rather than a side-by-side point estimate, supported an equivalence-style claim. Lang et al., Zerouali et al., and Galbusera et al. all reported weak TK agreement, with Galbusera et al. explicitly concluding that TK was not clinically reliable [25,26,27]; Kim et al. found the tightest kyphosis agreement, attributed to a large, high-quality training set [17]. For cervical parameters, Vogt et al. was the only study directly comparing automated and manual measurement pre- and postoperatively, finding strong C2-C7 SVA agreement but weaker T1 slope agreement, with T1 slope detected in only 48-51% of cases due to cervicothoracic overlap [8]. For coronal Cobb angle, Caesarendra et al. and Zerouali et al. found automated agreement exceeding inter-human agreement [26,28]; Lang et al. found automated Cobb MAD substantially better than inter-rater human MAD [25].

Postoperative and Instrumented Spines

Across the four studies evaluating both timepoints, manual inter-rater reliability remained essentially stable across the surgical divide while automated agreement consistently deteriorated [8,22,29]. Löchel et al. demonstrated this most starkly: human ICC remained ≥0.99 postoperatively while automated PI/SS ICC declined to 0.71-0.85, with detection rate falling to 84.8% [22]. Vogt et al. found a more favourable postoperative picture for cervical SVA [8]. Almahozi et al. found perfect automated intraobserver reproducibility (ICC 1.00) regardless of instrumentation status, despite clear accuracy bias - a dissociation between reproducibility and accuracy worth noting explicitly, since the two properties should not be conflated when judging clinical readiness [19].

Clinical Decision Thresholds and Inferred Surgical Planning Impact

No included study directly measured a surgical-planning outcome; the following reflects an inferred impact, derived by comparing measurement discrepancies against established correction thresholds (PI-LL >10°, SVA >40 mm). Kim et al. noted that wide LoA for LL and PI could shift an individual patient's measurement enough to misclassify PI-LL mismatch severity [17]. Orosz et al. found postoperative LL RMSE could push borderline patients across the 10° threshold [29]. Almahozi et al. explicitly cautioned against using individual automated measurements for threshold-dependent surgical decisions given wide LoA for LL and SVA, while acknowledging value for population-level screening [19]. Galbusera et al. concluded most parameters were not yet clinically usable for surgical planning, the exception being PT, which approached manual precision [27].

Discussion

Summary of Evidence

Automated machine vision systems can measure spinal sagittal balance parameters with accuracy approaching that of experienced clinicians, but this varies substantially by parameter, patient complexity, and surgical context. SVA and PT were the parameters where automated measurement most consistently matched, and in reproducibility terms occasionally exceeded, manual measurement. Automated SVA ICC values of 0.99-1.00 were replicated across architecturally distinct systems and diverse populations [17,23,24,25]. PT similarly achieved ICCs of 0.96-0.99 across multiple cohorts [16,17,22,25], with Kim et al. reporting automated PT MAE of only 0.4°, numerically exceeding inter-surgeon PT agreement in the same study [17]. By contrast, thoracic kyphosis and pelvic incidence in complex deformity cohorts were substantially harder for automated systems to match. TK ICC fell to 0.58-0.69 in biplanar EOS cohorts [25,26], well below human inter-rater ICC exceeding 0.90 in the same studies, and Galbusera et al. concluded that automated thoracic kyphosis was not clinically reliable in their model (SEM 8.6°, mean difference −7.6°) [27].

A recurring finding is that mean accuracy does not capture individual-case error distribution. Several studies reported clinically acceptable mean absolute errors for LL (1.69°-3.0°) [15,16,17] alongside Bland-Altman limits of agreement spanning 15°-25° for the same parameter [17,20], meaning an individual borderline patient's automated measurement could differ from the surgeon's value by a margin large enough to reclassify PI-LL mismatch status. Several systems also showed systematic directional bias: Cho et al. found LL overestimation in 26 of 42 cases [18], Lee et al. found LL underestimation (bias −3.42° to −4.28°) [20], and Almahozi et al. found overestimation bias of 6.31° for LL and 3.95 mm for SVA [19]. Where a source study performed a specific paired test, Yuan et al. [15]: p = 0.58; Harake et al. [16]: p = 0.24-0.93; Korez et al. [21]: paired t-test p > 0.05 across five pelvic parameters, that test did not detect a difference from manual measurement in that sample; this is a narrower and more defensible claim than describing automated measurement as generally "equivalent," since a single non-significant result does not itself demonstrate clinical interchangeability.

Postoperative performance data add a further dimension. Across the four studies evaluating both timepoints, manual inter-rater reliability remained essentially unchanged across the surgical divide while automated agreement consistently deteriorated [8,22,29]. Löchel et al. demonstrated this most starkly: human inter-rater ICC remained ≥0.99 postoperatively while automated ICC for PI and SS declined to 0.71-0.85, with postoperative RMSE reaching 10.2° for PI and 11.6 mm for SVA [22]. Almahozi et al. additionally found perfect automated intraobserver reproducibility (ICC 1.00) regardless of instrumentation status, even as accuracy relative to manual measurement showed clear systematic bias [19], a reproducibility/accuracy dissociation discussed further below.

Failure rates ranged from 0% [16,20,23,27] to 76.2% of patients having at least one failed parameter [19]. Lang et al. found complete automated segmentation in only 45% of test cases, attributing failures to severe pathology, high BMI, and foreign objects - precisely the complex cases where automation would be most valuable if it worked [25]. Processing speed showed the clearest and most consistent automated advantage: reductions of approximately 139-fold (Hoppe et al.) [23], 192-fold and 238-fold against specialist and junior-resident measurement respectively [26], and a statistically confirmed 3.6-fold reduction for the commercial Brainlab platform (t(20) = −9.952, p < 0.001) [19]. Korez et al. reported sub-one-second automated processing against 75 seconds manual processing, plus a 30-minute software tutorial requirement for manual measurement [21].

This review's principal strengths are its explicit benchmarking of automated error against same-study human reliability rather than an arbitrary absolute threshold, its treatment of case-level failure as a discrete outcome, and its dedicated postoperative/instrumented-spine analysis, each addressing a gap in the three prior reviews on this topic [10-12].

Limitations

This review has several limitations. First, no protocol was prospectively registered on PROSPERO, and inter-rater agreement statistics (e.g., Cohen's kappa) were not calculated for title/abstract screening. Second, the search was restricted to four databases plus a Google Scholar supplement capped at 200 relevance-ranked (not date-sorted) results, and non-English-language studies were excluded, so some eligible literature-particularly recent or non-English work-may have been missed. Third, quantitative meta-analysis was not performed for any parameter, including SVA and PT, despite tightly clustering ICC values (0.96-1.00) across four or more studies; findings are therefore descriptive rather than pooled, and no formal statistical test (e.g., confidence-interval overlap or difference-in-differences) was used to compare AI-versus-human error, limiting the strength of "comparable to human reliability" claims. Fourth, 11 of 16 included studies were rated high risk of bias on QUADAS-3, predominantly from non-consecutive sampling and unincorporated case exclusions, and the post hoc sensitivity check restricted to lower-risk studies was narrative rather than quantitative. Fifth, patient-level (versus image-level) separation between AI training/validation/test partitions, external-site validation, and algorithm-version reporting was unconfirmed across all 16 studies. Sixth, "failure" was heterogeneously defined across source studies and only partially reclassifiable into the four-tier hierarchy proposed here; human-corrected and clinically unacceptable-output tiers could not be reliably extracted. Seventh, no included study directly measured a surgical-planning outcome, so threshold-based clinical impact is inferred, not observed. Finally, a formal certainty-of-evidence framework (GRADE) was not applied, and no funnel-plot or other publication-bias assessment was feasible without pooled estimates.

Implications

The mechanistic explanation for the SVA/PT-versus-TK/PI asymmetry above is plausibly attributable to greater anatomical variability and curvature ambiguity in thoracic endplate identification, where rib overlap and lower image contrast compound landmark-detection error, precisely the zones where experienced clinicians draw on contextual anatomical reasoning that current architectures cannot fully replicate [21,22,25]. This interpretation, if correct, implies automated systems should not be deployed as monolithic entities: selective reliance on automated output for SVA and PT, with human verification retained for TK and PI in complex cases, represents a clinically rational hybrid approach.

A consistent directional bias, even a modest one, is arguably more clinically hazardous than equivalent random error, since it shifts an entire population of measurements in the same direction relative to a surgical threshold rather than scattering cases on either side of it [19,29]. The reproducibility/accuracy dissociation seen in Almahozi et al. is similarly underappreciated in this literature; the two properties should not be conflated when judging clinical readiness [19]. The tendency for automated systems to fail preferentially on the most anatomically complex cases [25] also represents a survivorship-bias concern for this literature as a whole: reported accuracy reflects only the cases a system did not give up on, and the hardest cases, where automation would be most valuable, are disproportionately absent from the denominator.

Synthesising the documented parameter-, population-, and context-dependence, a practical framework for clinical deployment can be proposed. SVA and PT may be considered high-confidence parameters in preoperative, non-instrumented populations resembling the validated cohorts, where automated outputs may reasonably be used with routine rather than case-by-case verification. LL and PI may be considered for conditional use, particularly when automated measurements fall close to clinically relevant surgical correction thresholds, such as PI-LL >10°, where directional bias could plausibly alter threshold classification. TK, SS, and measurements involving difficult cervical landmarks may warrant human verification because of their greater variability and dataset-dependent agreement. Human oversight should remain mandatory in postoperative and instrumented cases, severe deformity, transitional anatomy, poor-quality radiographs, and situations in which the algorithm generates a low-confidence output. This framework represents a synthesis of the findings of the present review rather than a validated clinical decision tool and should therefore be prospectively evaluated before routine clinical implementation.

## Conclusions

Machine vision-based automated measurement of sagittal balance is a promising and efficient alternative to manual measurement in preoperative spinal deformity assessment, with several mature architectures achieving accuracy for key parameters, particularly SVA and PT, comparable to experienced manual measurement under the specific conditions tested. By contrast, available evidence suggests that automated measurement performance is less robust in postoperative and instrumented spines, particularly in the presence of extensive instrumentation and complex anatomical alteration, where human adaptability to imaging artefacts cannot yet be replicated algorithmically, and case-level failure rates and systematic biases in some platforms further limit readiness for standalone surgical-planning use. Because no included study measured a surgical-planning outcome directly, this limitation is inferred from measurement-discrepancy-versus-threshold comparisons rather than observed clinically. Until standardised validation frameworks and prospective postoperative studies establish consistent reliability across patient populations, automated sagittal balance analysis should be regarded as a valuable adjunct to, rather than a replacement for, manual clinical measurement in spinal deformity care.

## Appendices

**Table 3:** Characteristics of the studies included.

Author(s) and year	Study design	Population type and N	Mean age (years)	AI/ML intervention (method)	Control/comparator	Segmentation performance	Success/failure rate	Speed/efficiency
Cho BH et al. (2020) [16]	Cross-sectional database study	Patients undergoing lateral lumbar X-ray. Training/validation: n = 629. Test: n = 151. Surgeon comparison: n = 50.	Not reported	U-Net CNN for vertebral body segmentation + CV algorithm Landmark: L1 and S1 identification → superior endplate → LL Augmentation: 629→12,580 images Platform: Python 3.5, Keras 2.2.0, NVIDIA GTX 1080-TI	Manual by three surgeons (chief resident, spine fellow, attending surgeon). Gold standard: Attending surgeon. Each was measured twice over a two-week period.	Training DSC: 0.966. Validation DSC: 0.923. Test DSC: 0.821. AUC (ROC): 0.914. Accuracy: 0.862. Training time: ~24.4 hours.	127/151 (84.1%) LL measured. Surgeon subset: 42/50 (84%). Failure = inability to identify L1 or S1 ~16% failure rate	0.14 seconds/radiograph
Korez R et al. (2020) [19]	Retrospective diagnostic accuracy study	Non-consecutive adults, N = 55 (21 M, 34F). 97 sagittal X-ray images. Include pre- and postoperative images (instrumentation/hip prostheses).	Mean: 69.6 years (range: 35–85)	Deep learning: two CNNs. 1) RetinaNet: bounding box detection (femoral heads, sacral endplate, C7, C1, C6, T6, T12, L5). 2) U-Net: landmark regression (anterior, center, posterior S1 endplate). Parameters: SS, PT, ST, PI, SSA. Handles superimposed femoral heads, occluded C7, dome-shaped sacrum.	Manual by one spine surgeon (M.P.) using the SurgiMap Spine Manual. Reliability: MAD 4.1°(SS), 1.7°(PT), 4.3°(PI). Avg manual time: 75s + 30-min tutorial.	Bounding box via RetinaNet; landmark probability maps via U-Net. Not reported as DSC.	96/97 images processed (1 excluded: very poor quality). SS: 96 images; PT and PI: 90 images (femoral heads not visible in 6); ST and SSA: 92 images (C7 not visible in 4). DL tool: ≤1 sec for all eligible images.	≤1 second per image vs. 75 seconds manual + 30-min tutorial
Kim YT et al. (2023) [15]	Retrospective diagnostic accuracy/pipeline development study	No prior surgical treatment. N = 1,810 unique patients. Total radiographs: 2,007. Train: 1,607 / Val: 200 / Test: 200.	Not explicitly reported (adult patients, implied by SRS-Schwab ASD classification)	Mask R-CNN (instance segmentation) + CV pipeline. Classes: C7, T4–L5, sacrum, femoral heads. Backbone: ResNeXt-101(32×4d)-FPN. Preprocessing: CLAHE. CV (OpenCV): findContours + approxPolyDP, DP algorithm. Parameters: TK, TLK, LL, SS, PT, PI, SVA.	Three surgeons: Criterion standard: Neurosurgeon >20 years. Surgeon 1: Neurosurgeon >10 years. Surgeon 2: Neurosurgeon >10 years. Manual on 200 test images.	AP50: 96.2%. AP75: 75.7%. Overall AP: 64.9%. Overall AR: 70.4%. DSC: 92.6%. Class AP50: C7 = 54.8%, T4–L5 = 81.5%, sacrum = 77.5%, femoral heads = 73.0%.	Six segmentation failures excluded (one T4 artifact, five sacrum/L5 overlap); six sacralization/lumbarization cases excluded. Total excluded: 12/200 (6%).	Training: 36 epochs on 2× RTX 2080 TI. Inference time not explicitly reported.
Zerouali M et al. (2023) [24]	Retrospective observational study	N = 100 patients (35 M, 65 F). Whole-spine radiographs, standing, Jan- Jul 2022. Total dataset: 932 (Train: 748 / Val: 94 / Test: 90).	Median: 14 years (IQR: 13–15.25). Range: 3–64 years.	Mask R-CNN (ResNet50-FPN) instance segmentation. Three model variants: HL256, HL512, HL1024. Coronal: Cobb angle, scoliosis, pelvic asymmetry. Sagittal: TK, LL, SS, PT, PI, C2I, C2S, C27L, T1I, T1S, L1I, C2-7 SVA, C7 SVA (13 total).	Junior radiologist measurements approved/adjusted by a senior MSK radiologist (ground truth). Specialist vs. resident also compared.	Best model (HL512): detection AP0.75 = 0.73. Segmentation AP0.75 = 0.706. IoU threshold: 0.80. Three validation + seven test cases failed (all models).	72/100 (72%): AI output reliable with NO modification. Overall failure: three validation + seven test cases.	Average: 3.8 s (SD 0.8s) per full parameter extraction vs. Specialist: 732.4 s vs. Resident: 904.15 s.
Vogt S et al. (2024) [8]	Retrospective monocentric cohort study (validation). Pre- and postoperative.	N = 129 patients, anterior cervical surgery (ACDF/CDA). Degenerative cervical spine disease. Training set: 519 images, 18 clinical sites.	53 years (84 F, 45 M)	Four interlinked deep CNNs: 1) Mask-RCNN (segmentation); 2. C1 landmark detection (UNet); 3. C2 landmark detection (UNet); 4. C3–T1 landmark detection (UNet). Parameters: C2–C7 lordosis, C1–C7 SVA, C2–C7 SVA, C7 slope, T1 slope DICOM input, PyTorch/TensorFlow	Two experienced physicians (Raters 1 and 2). Each measured TWICE, blinded. Horos Viewer software. Intra- and inter-rater reliability assessed.	Dice score: Preop: 0.91. Postop: 0.87. C7 detected: 128/129 preop, 127/129 postop. T1 detected: 48% preop, 51% postop (low rate)	C1/C2/C7 detected: 99% preop, 98% postop; T1 slope: 48% preop, 51% postop; three total failures: C7 not detected	Not reported for inference. Training: 100 epochs per CNN (GTX 1080)
Almahozi A et al. (2025)[17]	Retrospective validation study (single center)	Adults with degenerative spinal disease (instrumented and non-instrumented); N = 21	Not reported	Brainlab Elements Spine Planning Software Automated vertebral body identification + spinopelvic parameter generation from EOS biplanar stereoradiographs. Automated labeling confirmed/adjusted by the user	Manual measurements using PACS (MERLIN, Phoenix-PACS); eight observers (two experienced spine surgeons, three senior + three junior neurosurgical residents); two rounds, one-week interval, blinded	ICC (automated) = 1.00 (perfect, all rounds/observers). Manual ICC: intraobserver 0.44–0.99; interobserver 0.53–0.99. Lowest interobserver ICC: PTC = 0.53, MTC = 0.67.	Failure rate: SVA: 5/21 (23.8%); CB: 8/21 (38.1%); PTC: 11/21 (52.4%); MTC: 5/21 (23.8%); LC: 2/21 (9.5%). Coronal misclassified as sagittal: two cases ≥1 parameter failure: 16/21 (76.2%). Overall: 39/189 measurements failed (20.6%). Success rate: 79.4%.	Automated: 62±27 s (R1), 58±21 s (R2). Manual: 227±72 s (R1), 221±65 s (R2). 3.6× faster (p < 0.001).
Caesarendra W et al. (2022) [26]	Prospective/retrospective diagnostic accuracy study (single-center + public dataset, open access)	Adolescent Idiopathic Scoliosis (AIS) Public: AASCE MICCAI 2019 dataset Local: AIS clinic patients (Nov 2018–Sep 2020). Total X-rays: 551 (Train: 481 / Test: 70).	Not reported	ACAMM: Automated Cobb Angle Measurement Method CNN (152-layer ResNet backbone) detects 68 landmarks (four corners + center × 17 vertebrae). Focal loss; Adam optimizer (LR = 0.0001). Input: AP standing X-rays (C7 to femoral heads). Stages: LBP + cascade classifier → CNN vertebrae detection → Cobb angle calculation.	Two neurosurgeons (blinded): Observer 1: scoliosis specialist (reference standard). Observer 2: non-specialist. Both used PACS in-built measurement tools.	Accurately detected 17 vertebrae (C7–sacrum). Robust across varying contrast, lighting, body habitus. Handles severe scoliosis (up to 79° Cobb). Missing one to two vertebrae compensated by curve fitting.	Success rate: 69/70 (98.6%) within ±5° of reference. Only 1/70 (1.4%) outside ±5° (CA>40° group). Robust to poor contrast and severe curves.	Not explicitly reported; GPU-based (RTX2060) inference significantly faster than manual (Inference time not numerically quantified).
Hoppe et al. (2025) [21]	Retrospective diagnostic accuracy multi-reader study (295 train/val; 65 test images)	Consecutive patients, lateral full-spine standing radiographs (EOS) N (test set): 65 patients (39F, 60.0%). Pathology: 35.4% vertebral fractures; 24.6% prior instrumentation. Age range: 7–85 years.	47.8 ± 24.1 (range 7–85). All age groups.	Two-stage R-CNN: Stage 1: detects whole-spine/vertebral centers. Stage 2: detects 4 corner landmarks per vertebra (C2–S1; ~94 labels/case). Geometric post-processing → TK, LL, SS, SVA.	Six human readers: three radiologists (three-, six-year exp.); three orthopedic surgeons (three-, five-,eight-year exp.). Reference standard = median of all six readers.	Landmark-detection based (not pixel segmentation): 100% of 65 test cases successfully processed (incl. fractures, deformity, instrumentation).	100% (65/65). All errors below <5° clinically acceptable inter-reader variability threshold.	AI: ≤1 sec/case. Human: 139 sec/case (mean; range 86–231 sec) ~139× faster than the average human reader.
Weng et al. (2019) [22]	Retrospective algorithm development + validation (990 train/eval; 99-image reader subset).	Patients with degenerative changes or spinal deformities, standing lateral whole-spine radiographs. N (total): 990 patients. 13% <12y; 42% 13–18y; 45% >18y N (reader subset): 99 images; 23% with spinal implants.	Not reported as mean ± SD. Stratified by age band.	31-layer ResUNet (CNN, keypoint/heatmap detection). Predicts two landmarks: C7 center and posterior corner of sacrum via exponential heatmaps (σ = 1.57 optimal). SVA = horizontal distance between the two predicted landmarks.	Three human annotators (99-image subset): senior resident; orthopedic surgeon (>5 yrs); junior resident; orthopedic surgeon; radiologist. Full 990-set labeled by a senior resident (consensus with senior spine surgeon for difficult cases).	5-mm detection rate: C7 center: 91.1% (±1.2%). Sacrum corner: 87.1% (±0.4%) 1-mm detection rate: C7 49.6%; sacrum 32.8%. Best: 31-layer ResUNet + exponential heatmap.	Outlier rate (>10 mm error): 6.87% of cases. Narrow Gaussian heatmaps (σ = 0.79) highly unstable (outlier rate up to 59.4%). No binary case-level pass/fail reported.	AI: ~0.2 sec/case (SVA calculation). Human reading time not directly reported.
Yuan et al. (2024) [13]	Retrospective model development + validation (1,240 images; 8:1:1 train/val/test)	Adult outpatients, standing lateral lumbar radiographs. N (total): 1,240 (from 2,853 screened). Train: 992 / Val: 124 / Test: 124. Exclusions: prior spine surgery, severe deformity, metabolic bone disease, fracture, TB, tumor, poor image quality.	Train: 52.46 ± 16.54; Val: 50.85 ± 16.16; Test: 51.79 ± 17.23 (M:F ≈ 1:1).	RADMFNet — segmentation network with dilated convolution, ResNet backbone, multi-scale attention (SE-ResNet), multi-scale feature fusion; Harris corner detection → 10 vertebral key points. Parameters: LL, SHA, ISA(L4–L5/L5–S1), PLS(L4–L5/L5–S1).	Three spine surgeons (S1–S3), independently annotated all 1,240 images. Reference standard = mean of three surgeons' measurements. Intraobserver reliability: S1 re-annotated the test set after one month.	Lumbar Dice: 0.962; accuracy: 0.947; sacrum Dice: 0.954; accuracy: 0.939; outperformed UNet (0.937), Att-UNet (0.946), UNet3+ (0.947), TransUNet (0.937); Key-point PCK (≤3 mm): 88–98% across 10 landmarks.	Not reported as binary case-level success/failure. All images processed PCK @≤3 mm: 88–98%.	Processing speed/time not explicitly quantified (implied efficiency gain over manual measurement).
Orosz et al. (2022) [27]	Retrospective validation study (600 images for AI training; 200 images/100 pts for validation)	Patients undergoing lumbar spine fusion, pre- and post-op standing lateral lumbar radiographs. N (validation): 100 patients / 200 radiographs (pre + post). N (AI training): 600 radiographs, 18 clinical sites. Pathology: 71% lumbar segmental instability; 43% anatomical variance.	Not reported as mean ± SD. Cohort = 100 lumbar fusion patients, 2017–2019.	Three parallel deep learning models (TensorFlow): Model 1: Cascade R-CNN — segments lumbar vertebrae, sacrum, femoral heads, instrumentation. Models 2–3: U-Net-based landmark placement (6 pts/vertebral body; 5 pts on sacrum). Outputs: LL, PI, PT, SS via line regression/slope computation.	Rater 1: institutional expert, all 200 radiographs. Rater 2: one of four fellowship-trained spine surgeons, 50% subset (100 radiographs). AI and humans blinded to each other.	Dice coefficient: 0.85 (instance segmentation, 60 test images). Avg anatomical structure detection rate: 96%. Landmark Euclidean error: L1 endplate 2.1 mm; sacral endplate 1.9 mm.	98% preoperative, 95% postoperative. Failures: undetected L1/femoral heads (preop); transitional anatomy, midpositioned sacrum, undetected femoral heads (post-op).	Not explicitly quantified (sec/case). Framed as time-saving vs. manual digital measurement workflow.
Harake et al. (2024) [14]	Retrospective model development + validation (761 train/val; 40-image held-out test set),	Patients undergoing standing sagittal whole-spine radiographs (C7–S1 + femoral heads), 2018–2023, N (train/val): 761 radiographs, N (test): 40 radiographs, labeled by four reviewers. Pathology: 73% DDD, 58% scoliosis, 45% stenosis; 58% instrumentation; 7.5% transitional variants.	Not reported as a single cohort mean ± SD	SpinePose: three parallel CNNs (Keypoint R-CNN, PyTorch). L1 model and S1 model: top-down region-based (box detection → keypoints). R model: bottom-up encoder-decoder for remaining keypoints (C7, T1, femoral heads). Parameters: SVA, PT, PI, SS, LL, T1PA, L1PA.	Four reviewers: R1 (ground truth): fellowship-trained spine surgeon, 18 years. R2: fellowship-trained spine surgeon, 5 years. R3: fellowship-trained neurointerventional radiologist. R4: senior neurosurgery resident.	Keypoint-based (PCK metric, not Dice). PCK @5 mm: L1/S1 landmarks, 75–93%; remaining landmarks, 38–60%. PCK @10 mm: nearly all successful, 88–100% 3-CNN SpinePose outperformed single bottom-up model (e.g., LL median error 4.3° vs. 6.5°)	Not framed as case-level detection-failure rate Model produced predictions for all 40 test images. Reliability reported via ICC/PCK.	Not explicitly quantified (sec/case). Framed as eliminating need for manual landmark entry vs. prior semi-automated tools.
Löchel et al. (2024) [20]	Retrospective validation study (946 training images; 141 patients / 243 images tested)	Adult spinal deformity (ASD) patients, long-construct instrumentation (>3 segments). N (test): 141 patients — 118 preop + 125 postop. N (training): 946 images, 22 clinical sites. 21 patients with cement-augmented screws postop.	Not reported as mean ± SD. ASD population	Three-step deep learning pipeline: 1) Mask-RCNN segmentation (cervical/thoracic/lumbar vertebrae, sacral endplate, femoral heads, instrumentation). 2) U-Net landmark detection (5-point sacrum; six-point L1) via heatmap regression. 3) Line-fitting/regression → SS, PT, PI, LL (L1–S1), SVA. Training: 100 epochs, NVIDIA GeForce 1080.	Two human raters (F.A. and J.L.), SurgiMap Spine software. Each rater's measurements repeated for intra-observer reliability. Ground truth = manual SurgiMap measurements.	Mask-RCNN-based (qualitative only; no Dice reported). Subgroup analysis: no significant accuracy difference with implants/cement augmentation (p > 0.05).	91.5% preoperative, 84.8% postoperative (lowest postop detection rate among all studies; attributed to long-construct instrumentation artefacts),	Not explicitly quantified (sec/case), Positioned as enabling efficient analysis of large databases,
Lang S et al. (2024) [23]	Retrospective diagnostic accuracy study. Single institution.	N = 393 patients. 555 bi-planar radiographs. Spinal deformity (AIS, ASD). 58/100 test-set = scoliosis patients. Uninstrumented only. Age range: 8–92 years. EOS imaging system.	Mean 42.2 ± 24.3 years (wide range incl. paediatric)	Modified U-Net with inception modules TensorFlow / Python 4 ROI segmentation networks (neck, spine, sacrum, femur). Binary cross-entropy loss; Adam optimizer (lr = 0.0005). 500 epochs, batch size 20. Biplanar AP + lateral radiographs. 232 anatomical landmarks per image. Parameters: Cobb°, THK°, LL°, SVA, SS°, PT°, PI°.	Blinded medical expert using mediCAD software. Inter-rater: three experienced clinicians (ICC on n = 10). Intra-rater: one expert, repeated ×3 at three-day intervals. Reference: manual annotation by clinicians and medical students.	Preprocessing success: 533/555 (96%). Complete segmentation (all ROIs): 45/100 (45%).	Overall complete prediction: 45/100 (45%). Frontal (Cobb) only: 61/100 (61%). Scoliosis subset: 34/58 (58.6%). Parameter-wise: THK 69%; LL 58%; SVA 86%; SS 85%; PT 84%; PI 84%. Failure causes: severe pathology, high BMI, foreign objects.	Not reported. No processing time data provided. GPU: NVIDIA RTX A6000.
Lee S-B et al. (2025) [18]	Retrospective diagnostic accuracy. Single institution. Catholic Kwandong Univ., South Korea 2019–2021.	N = 888 lateral X-rays (from 2,397 screened). Whole-spine lateral radiographs. General spinal pathology. Train: 740 / Val: 98 / Test: 50. ~49% male / 51% female. Age range: 3–89 years.	Train: 48.24 ± 25.36. Val: 48.11 ± 25.76. Test: 48.7 ± 24.78.	Keypoint R-CNN + FPN. Backbone: ResNeXt-101 (32×8d). Framework: Detectron2 (Facebook AI). Pre-trained ImageNet weights; 30,000 training iterations. Optimizer: SGD; Input: 1024px. Augmentation: rotation, brightness, saturation; 4 keypoints only: sup-ant and sup-post of L1 and S1. Parameter measured: LL angle ONLY.	Two orthopedic surgeons (>10 years' experience each). Independent manual annotation of 50 test images. No specific software named.	Keypoint detection (mAP): Bounding box. mAP Avg: 71.63 / Best: 75.78. Keypoint mAP (OKS). Avg: 86.61 / Best: 92.63. Four keypoints only (L1 and S1 landmarks). No full vertebral segmentation.	50/50 test images processed (100% completion). No cases of failure or exclusion from the test set. Note: 1,509/2,397 images excluded pre-training for quality.	Not reported. No inference time given. Hardware: AMD Ryzen Threadripper PRO + Dual NVIDIA RTX 4090.
Galbusera F et al. (2019) [25]	Retrospective diagnostic accuracy. Single institution: IRCCS Istituto Ortopedico Galeazzi, Italy.	N = 493 patients; 493 biplanar EOS radiographs; AIS, adult scoliosis, degenerative disorders, spinal stenosis; 48 patients with spinal instrumentation. Train: 443 / Test: 50.	Not reported. No mean age stated.	FCN + DSNT layer (Differentiable Spatial to Numerical Transform) Framework: Keras / TensorFlow seven-layer fully convolutional network; 78 separate localizer models (one per landmark per plane); 39 landmarks × 2 planes (coronal + sagittal). Parameters: T4–T12 kyphosis, L1–L5 lordosis, Cobb, PI, SS, PT. GPU: NVIDIA Titan Xp.	sterEOS software (3D spinal reconstruction). Manual 3D reconstruction by clinicians as ground-truth 3D coordinates of 39 landmarks from sterEOS reconstructions via a custom C++ program. No additional human rater comparison reported.	No F1/Dice/mAP reported. Qualitative: spine shape 'perceptually convincing in all cases'. Known failure zones: T1 and S1 endplates; coronal S1 orientation. 78 localizer models trained separately.	All 50 test cases processed (100% completion). Qualitatively successful, including instrumented spines (n = 48). Known failure zones: coronal S1 orientation (gross errors in subset); T1 endplate reduced accuracy. No formal % failure rate defined.	Not reported. No inference time given. Hardware: Linux workstation, NVIDIA Titan Xp.
